# RGMa collapses the neuronal actin barrier against disease-implicated protein and exacerbates ALS

**DOI:** 10.1126/sciadv.adg3193

**Published:** 2023-11-22

**Authors:** Mikito Shimizu, Naoyuki Shiraishi, Satoru Tada, Tsutomu Sasaki, Goichi Beck, Seiichi Nagano, Makoto Kinoshita, Hisae Sumi, Tomoyuki Sugimoto, Yoko Ishida, Toru Koda, Teruyuki Ishikura, Yasuko Sugiyama, Keigo Kihara, Minami Kanakura, Tsuneo Nakajima, Shuko Takeda, Masanori P. Takahashi, Toshihide Yamashita, Tatsusada Okuno, Hideki Mochizuki

**Affiliations:** ^1^ Department of Neurology, Neuroscience, Osaka University Graduate School of Medicine, Suita, Osaka, Japan.; ^2^ Department of Clinical Research, National Hospital Organization Osaka-Minami Medical Center, Kawachinagano, Osaka, Japan.; ^3^ Department of Neurotherapeutics, Neuroscience, Osaka University Graduate School of Medicine, Suita, Osaka, Japan.; ^4^ Department of Neurology, Higashiosaka City Medical Center, Higashiosaka, Osaka, Japan.; ^5^ Graduate School of Data Science, Shiga University, Hikone, Shiga, Japan.; ^6^ Department of Health Sciences, Neuroscience, Osaka University Graduate School of Medicine, Suita, Osaka, Japan.; ^7^ Department of Geriatric and General Medicine, Neuroscience, Osaka University Graduate School of Medicine, Suita, Osaka, Japan.; ^8^ Department of Clinical Gene Therapy, Neuroscience, Osaka University Graduate School of Medicine, Suita, Osaka, Japan.; ^9^ Osaka Psychiatric Research Center, Osaka Psychiatric Medical Center, Hirakata, Osaka, Japan.; ^10^ Department of Molecular Neuroscience, Osaka University Graduate School of Medicine, Suita, Osaka, Japan.

## Abstract

Repulsive guidance molecule A (RGMa) was originally identified as a neuronal growth cone–collapsing factor. Previous reports have demonstrated the multifunctional roles of RGMa mediated by neogenin1. However, the pathogenic involvement of RGMa in amyotrophic lateral sclerosis (ALS) remains unclear. Here, we demonstrated that RGMa concentration was elevated in the cerebrospinal fluid of both patients with ALS and transgenic mice overexpressing the mutant human superoxide dismutase1 (mSOD1 mice). Treatment with humanized anti-RGMa monoclonal antibody ameliorated the clinical symptoms in mSOD1 mice. Histochemical analysis revealed that the anti-RGMa antibody significantly decreased mutant SOD1 protein accumulation in the motor neurons of mSOD1 mice via inhibition of actin depolymerization. In vitro analysis revealed that the anti-RGMa antibody inhibited the cellular uptake of the mutant SOD1 protein, presumably by reinforcing the neuronal actin barrier. Collectively, these data suggest that RGMa leads to the collapse of the neuronal actin barrier and promotes aberrant protein deposition, resulting in exacerbation of the ALS pathology.

## Teaser

RGMa collapses neuronal actin barrier and promotes aberrant protein deposits, resulting in exacerbation of the ALS pathology.

## INTRODUCTION

Repulsive guidance molecule A (RGMa) is a glycosylphosphatidylinositol (GPI)–anchored membrane protein that belongs to the RGM family; it was originally identified as an axon guidance molecule in the visual system of the embryo (*1*). In adults, RGMa is expressed in not only neurons but also glial cells and exerts various functions under both physiological and pathological conditions (*2*). A variant of RGMa contains a C-terminal domain (variant C), which includes a high-affinity interaction site for Neogenin1 (NEO1), a receptor for RGMa (*2*). Binding of RGMa to NEO1 induces stabilization and dimerization of its ectodomain for activating the downstream signaling cascades (*2*). Recent reports have demonstrated that RGMa-mediated signaling plays major roles in not only axon guidance but also T cell activation (*3*, *4*), apoptosis induction (*5*), synapse formation (*6*), and angiogenesis (*7*). Notably, the inhibitory effects of RGMs on neurite growth rely on various signaling cascades downstream of NEO1, one of which is the regulation of small guanosine triphosphatases (GTPases) such as Ras, Rho, and Rac, thereby controlling actin dynamics (*2*, *8*, *9*).

These multifunctional roles of RGMa, as described above, have been demonstrated in both physiological and pathological conditions of the nervous system. For example, neutralizing antibodies against RGMa promoted recovery from impaired manual dexterity in monkey models of spinal cord injury (SCI) by promoting corticospinal tract fiber sprouting from the motor cortex (*10*) and improved functional recovery in a rat SCI model through axonal growth of the corticospinal tract (*11*, *12*). In addition, RGMa is greatly expressed in CD4^+^ T cells in experimental autoimmune encephalomyelitis mice, an animal model of multiple sclerosis (MS), and therapeutic inhibition of RGMa improved the clinical symptoms of diseased mice (*3*, *13*, *14*). RGMa up-regulation was reported in an animal model of neurodegenerative diseases, such as Parkinson’s disease (PD), in which anti-RGMa antibodies have therapeutic potential (*15*, *16*). These reports suggest that RGMa could be a therapeutic target for neurological disorders.

Amyotrophic lateral sclerosis (ALS) is a devastating neurological disease characterized by the accumulation of aberrant proteins in the motor neurons of the brain and spinal cord. Patients with ALS develop skeletal muscle weakness, resulting in death owing to respiratory paralysis that typically occurs 3 to 5 years following clinical onset (*17*, *18*). Although the precise pathological mechanisms remain elusive, several processes, including aberrant RNA metabolism (*19*–*21*), uncontrolled protein aggregation (*21*–*23*), neuroinflammation (*24*), altered control of apoptosis (*25*, *26*), and cytoskeletal dysregulation (*27*–*30*), are reportedly involved in ALS onset and progression. However, the role of RGMa/NEO1 signaling in ALS has not yet been fully investigated.

In this study, we demonstrated that altered RGMa/NEO1 signaling is involved in the pathogenesis of ALS. RGMa levels in the cerebrospinal fluid (CSF) were increased in not only patients with ALS but also an animal model of ALS. Neutralization of RGMa with a monoclonal antibody in transgenic mice overexpressing the mutant human superoxide dismutase1 gene (mSOD1 mice) improved their motor function and ameliorated their survival. In vitro analysis suggested that the inhibition of RGMa/NEO1 signaling suppressed the uptake of extracellular proteins through actin polymerization or by reinforcing the neuronal actin barrier. These data suggest a pivotal role of the RGMa/NEO1 signaling axis in the pathogenesis of ALS and furthermore indicate the use of RGMa as a therapeutic target in ALS.

## RESULTS

### RGMa is elevated in the CSF of patients with ALS and correlates with their clinical features

**Fig. 1.**
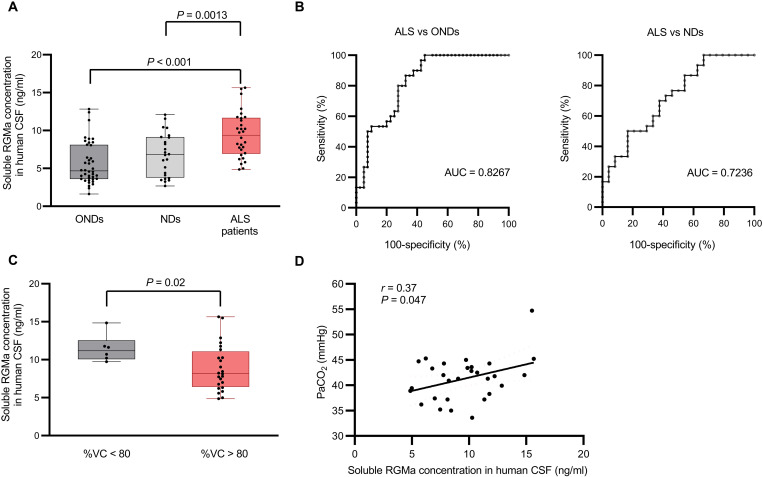
RGMa was significantly elevated in the CSF of patients with ALS. (**A**) RGMa in the CSF of patients with neurological disorders and control patients was measured using ELISA. RGMa was significantly increased in patients with ALS (*n* = 30) compared to those with NDs (*n* = 24) and ONDs (*n* = 40). (**B**) ROC analysis revealed that an AUC was high enough to distinguish ALS from NDs and ONDs. (**C**) The RGMa levels in the CSF were higher in patients with ALS with less than 80%VC than in those with more than 80%. (**D**) RGMa levels in the CSF were correlated with PaCO_2_ in patients with ALS. Error bars indicate mean ± SEM. RGMa, repulsive guidance molecule A; CSF, cerebrospinal fluid; ALS, amyotrophic lateral sclerosis; NDs, nonneurodegenerative disorders; ONDs, other neurodegenerative disorders; ROC, receiver operating characteristic; AUC, area under the curve; PaCO_2_, partial pressure of CO_2_ in arterial blood; %VC, vital capacity percentage.

To investigate the potential significance of RGMa in ALS, we measured RGMa levels in the CSF of patients with ALS with enzyme-linked immunosorbent assay (ELISA). The total RGMa levels in the CSF significantly increased in patients with ALS compared with those in patients with nonneurodegenerative disorders (NDs) and other neurodegenerative disorders (ONDs), as confirmed by analysis of covariance matching for sex and age (ALS versus NDs, *P* = 0.0013; ALS versus ONDs, *P* < 0.001) (Fig. 1A). In addition, the receiver operating characteristic (ROC) curve analysis of RGMa concentration demonstrated that RGMa was specifically elevated in the CSF of patients with ALS, raising the possibility that RGMa level in CSF could serve as a potential diagnostic biomarker for ALS [ALS versus ONDs: 95% confidence interval (CI), 0.59 to 0.86; *P* < 0.0001; ALS versus NDs: 95% CI, 0.73 to 0.92; *P* = 0.0073] (Fig. 1B).

Thereafter, we investigated the correlation between the clinical symptoms of patients with ALS and their RGMa levels in the CSF and found that patients with ALS with a lower vital capacity (%VC) (<80%) showed a higher RGMa level in the CSF than those with higher %VC (Fig. 1C). This result suggests that RGMa in the CSF is associated with respiratory dysfunction. Consistent with this observation, an arterial blood gas analysis of patients with ALS at initial hospitalization revealed that the partial pressure of arterial carbon dioxide (PaCO_2_) correlated with RGMa levels in the CSF (Fig. 1D, *r* = 0.37 and *P* = 0.047). Collectively, these data suggest that RGMa levels in the CSF of patients with ALS correlate with their prognosis, suggesting that RGMa in CSF could be a prognostic biomarker.

### The RGMa variant, which has a NEO1-binding domain, is elevated in the CSF of patients with ALS

**Fig. 2.**
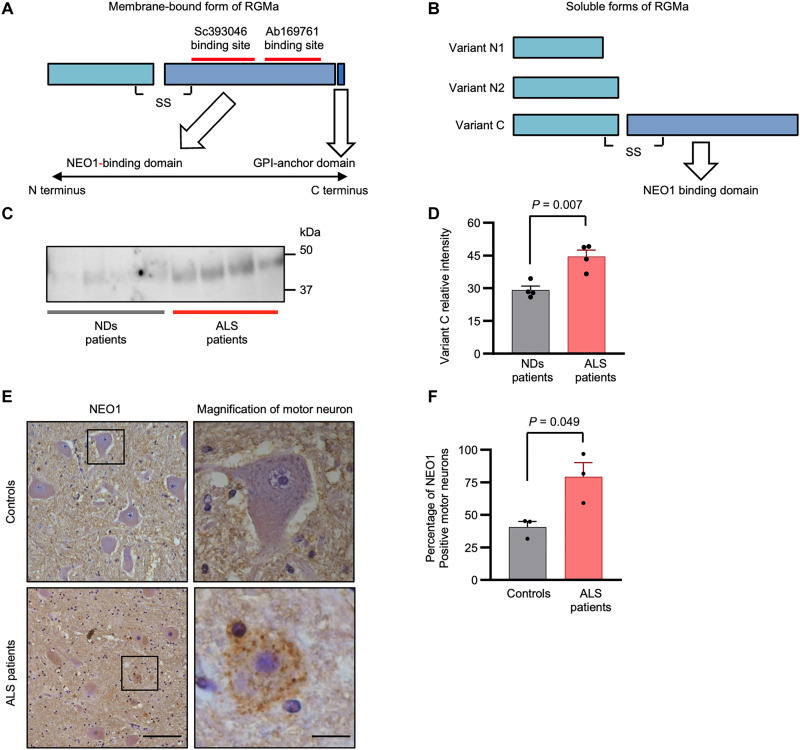
Variant C of RGMa, which contains a NEO1-binding domain, is elevated in the CSF of patients ALS. (**A**) RGMa is composed of two segments, N-terminal and C-terminal domain, connected with a disulfide bond. RGMa binds to NEO1 with the C-terminal domain, which contains GPI-anchor domain. Red lines indicate the binding sites of two antibodies (Sc393046 and Ab169761) used in co-immunoprecipitation and immunoblotting analysis, respectively. (**B**) Structures of three soluble forms of RGMa are shown. Variant that includes the C-terminal domain (variant C) had a strong potential for binding to NEO1. (**C**) Co-immunoprecipitation/immunoblotting analysis shows that variant C of RGMa was up-regulated in the CSF of patients with ALS (*n* = 4), compared to that of NDs (*n* = 4). (**D**) Quantification of the co-immunoprecipitation/immunoblotting analysis in (C). (**E**) Immunohistochemical analysis for NEO1 is shown. NEO1 was up-regulated on shrunken motor neurons in the spinal cords of patients with ALS (*n* = 3) compared to that of NDs (*n* = 3). Images are representative. (**F**) Percentage of NEO1-positive motor neurons in all the motor neurons in the spinal cords of patients with ALS and controls. Scale bars, 100 μm (left) and 12.5 μm (right). Error bars indicate mean ± SEM.

After shedding, the ectodomain of RGMa provides three forms of soluble fragments exerting their functions (*31*). These three forms consist of N1, N2, and C variants, the last of which contains a C-terminal domain, capable of binding to NEO1 and activating downstream signaling cascades (Fig. 2, A and B). Although our ELISA analysis revealed that the total RGMa level was elevated in the CSF of patients with ALS, it remains unclear whether the level of the variant C form, which has an NEO1-activating capacity, is elevated. We quantitatively evaluated variant C using an immunoprecipitation approach followed by immunoblotting to confirm whether variant C level is increased in the CSF of patients with ALS. The variant C levels were elevated in the CSF of patients with ALS (Fig. 2, C and D), suggesting that the increased variant C levels of RGMa play a pivotal role in activating the NEO1 signaling pathway in the central nervous system (CNS) of patients with ALS.

We subsequently attempted to identify the localization of NEO1 in the spinal cord of controls and patients with ALS by performing immunohistochemistry in the spinal cord (the fifth lumbar spine vertebrae). In controls, NEO1 immunoreactivity was rarely observed in motor neurons, but only faintly in the neuropiles. In contrast, diffuse punctate staining patterns of immunoreactivity for NEO1 were observed in the cell bodies of motor neurons in the autopsied ALS spinal cord (Fig. 2E), suggesting the subcellular localization of NEO1 to be altered under the pathogenic conditions of ALS. In addition, the ratio of the number of NEO1-positive motor neurons to the total number of motor neurons was higher in the ALS spinal cord than in the control spinal cord (Fig. 2F). These results suggest that the aberrant secretion of variant C of RGMa and ectopic expression of NEO1 in the motor neurons cause nonphysiological RGMa-NEO1 signaling, resulting in the pathogenesis of ALS.

### The dysregulated RGMa/NEO1 axis in patients with ALS is recapitulated in the animal model of ALS

**Fig. 3.**
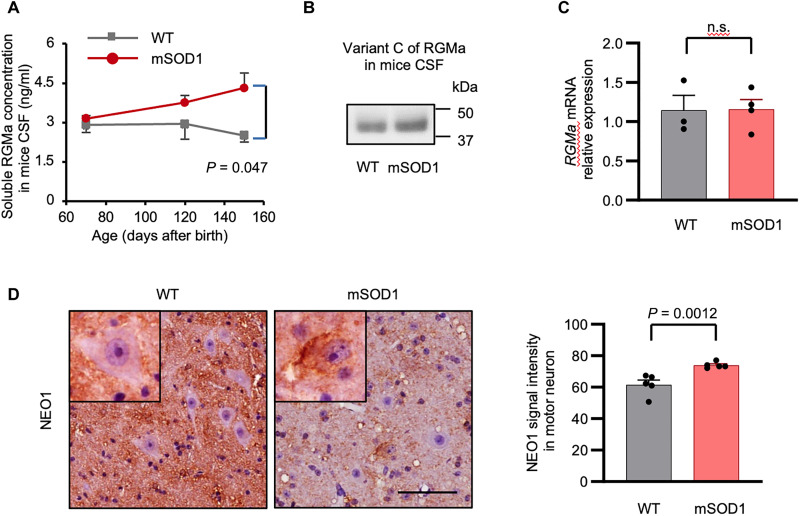
RGMa/NEO1 signaling axis was altered in mSOD1 mice. (**A**) ELISA revealed that RGMa levels increase over time in the CSF of mSOD1 mice (70 days old, *n* = 3; 120 days, *n* = 3; and 150 days, *n* = 4), compared to WT controls (70 days old, *n* = 3; 120 days, *n* = 3; ays150 days, *n* = 4). (**B**) Immunoblot analysis confirmed the presence of variant C (49 kDa) in the CSF of mSOD1 and WT mice, which strongly binds to NEO1. (**C**) Expression level of *RGMa* in the spinal cords was not different between the mSOD1 (*n* = 4) and WT mice (*n* = 3) at 105 days old in the qPCR analysis. (**D**) The subcellular localization of NEO1 in motor neurons was different between the mSOD1 and WT mice. NEO1 is mainly localized in the neuropile in the WT mice, whereas it is primarily expressed in the somas of motor neurons in the mSOD1 mice. Representative images are shown. Scale bar, 50 μm. Error bars indicate mean ± SEM. Ab, antibody; WT, wild-type; mSOD1 mice, transgenic mice overexpressing the familial ALS–associated G93A SOD1 mutation.

Considering that the variant C level of RGMa was elevated in the CSF of patients with ALS, we investigated whether this phenomenon could be recapitulated in an animal model of ALS. Therefore, we measured RGMa level in the CSF of mSOD1 mice. ELISA revealed a chronological increase in the total soluble forms of RGMa in the CSF of mSOD1 mice (Fig. 3A). Consistent with the data from patients with ALS, variant C of RGMa was detected in the CSF of mSOD1 mice by immunoprecipitation analysis (Fig. 3B). However, quantitative polymerase chain reaction (qPCR) analysis demonstrated no change in the expression level of *RGMa* mRNA in the spinal cord between mSOD1 and wild-type (WT) mice (Fig. 3C). Collectively, these data imply dysregulated secretion of RGMa in not only patients with ALS but also an animal model of ALS, suggesting altered protein processing of RGMa as the fundamental mechanism in ALS pathogenesis.

Immunohistochemical analysis for NEO1 was subsequently performed in the spinal cord of mSOD1 mice to examine the alteration of NEO1 expression in the neurodegenerative processes. NEO1 expression was up-regulated in the cell bodies of some anterior horn cells of mSOD1 mice, whereas in the WT mice, NEO1 was mainly expressed in the neuropile (Fig. 3D). These data indicate that the dysregulation of RGMa and altered distribution of NEO1 in motor neurons were recapitulated in the animal model of ALS.

### Anti-RGMa antibody treatment improves the motor function and lifespan of mSOD1 mice and promotes motor neuron survival through protein aggregate reduction

To evaluate the therapeutic potential of RGMa signaling in neurodegenerative processes in ALS, we investigated the effects of neutralizing antibodies against RGMa in mSOD1 mice. First, the anti-RGMa antibody concentration in the CSF of mSOD1 mice was measured to examine whether intraperitoneally administered anti-RGMa monoclonal antibody could cross the blood-brain barrier and enter the CNS compartment of mSOD1 mice. We confirmed that intraperitoneally administered anti-RGMa antibody was detectable in the CSF of mSOD1 mice (1.77 ± 0.55 μg/ml) (*n* = 3, mean ± SEM); the plasma concentration at steady state was 337 ± 45.03 μg/ml (*n* = 3, mean ± SEM).

**Fig. 4.**
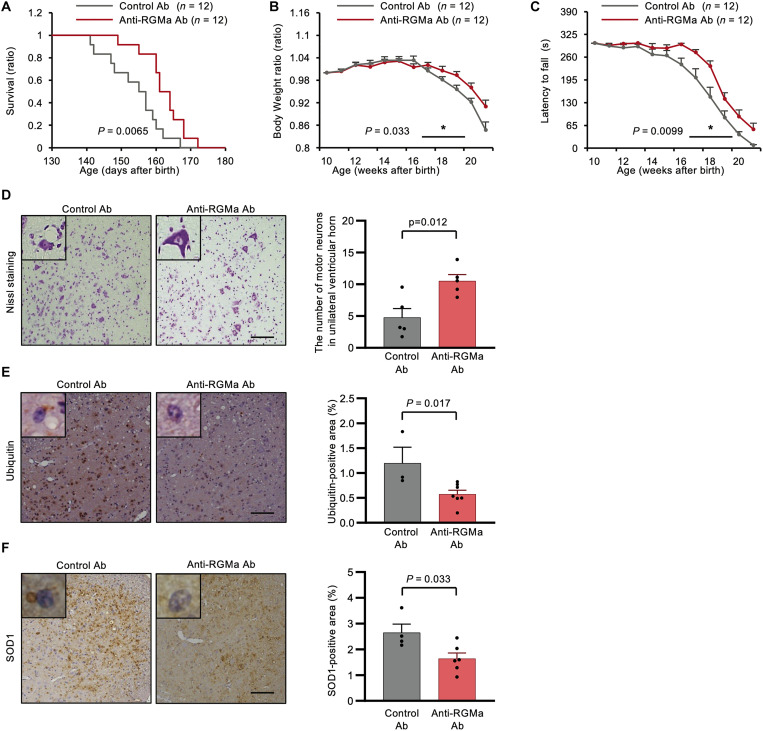
Anti-RGMa antibody had therapeutic potential in mSOD1 mice. (A to C) Clinical scores in the mSOD1 mice treated with anti-RGMa Ab (*n* = 12) and control Ab (*n* = 12) are shown. (**A**) Anti-RGMa Ab prolonged the survival of mSOD1 mice by a mean of 8.4 days (*P* = 0.0065). (**B**) Anti-RGMa Ab significantly mitigated body weight loss (*P* = 0.033), shown as the ratio to bodyweight in 10-week-old mice. (**C**) Anti-RGMa Ab in the mSOD1 mice improved their motor function measured by the rotarod test (*P* = 0.0099). (**D**) Nissl staining of the ventral horn of the spinal cord in mSOD1 mice is shown. Anti-RGMa Ab administration preserved the number of motor neurons with normal morphology (*n* = 5 for the control Ab–treated group and *n* = 5 for the anti-RGMa Ab–treated group). Representative images are shown. Scale bar, 100 μm. (**E**) Immunohistochemical analysis showed that anti-RGMa Ab treatment reduced the number of ubiquitin-positive neurons in mSOD1 mice (*n* = 3 for the control Ab–treated group and *n* = 7 for the anti-RGMa Ab–treated group). Representative images are shown. Scale bar, 300 μm. (**F**) Immunohistochemical analysis revealed that anti-RGMa Ab administration reduced the accumulation of SOD1 protein (*n* = 4 for the control group and *n* = 6 for the anti-RGMa Ab–treated group). Representative images are shown. Scale bar, 300 μm. Error bars indicate mean ± SEM.

Thereafter, we evaluated the therapeutic effect of the anti-RGMa antibody on neurodegeneration by measuring the body weight and motor function of mSOD1 mice (control antibody, *n* = 12 females; anti-RGMa antibody *n* = 12 females). Anti-RGMa antibody significantly extended the survival of mSOD1 mice by a mean of 8.4 days compared with the control mice [anti-RGMa antibody group (162.3 days) versus control antibody (palivizumab) group (153.9 days), *P* = 0.0065] (Fig. 4A). Anti-RGMa antibody administration significantly prevented body weight loss in 17- to 20-week-old mice (*P* = 0.033) (Fig. 4B) and ameliorated motor dysfunction in 17- to 20-week-old mice as evaluated by the rotarod test (*P* = 0.0099) (Fig. 4C). Nissl staining revealed that the number of anterior horn cells was significantly reduced in mSOD1 mice treated with control antibody compared with mSOD1 mice treated with anti-RGMa antibody. Moreover, the remaining anterior horn cells appeared atrophic in the mSOD1 mice treated with the control antibody (Fig. 4D). Intriguingly, ubiquitin-positive protein aggregates were decreased in the mSOD1 mice treated with anti-RGMa antibody compared with those in the control group (Fig. 4E). In addition, the anti-RGMa antibody reduced the aggregation of mutant SOD1 protein compared with the control antibody (Fig. 4F). On the other hand, Western blot analysis showed an increase in extracellular SOD1 protein in the CSF of mSOD1 mice compared to that in WT mice. However, treatment with anti-RGMa antibodies did not have an effect on the levels of SOD1 protein in the CSF (fig. S1, A and B). Immunohistochemical analysis (fig. S2A) and qPCR analysis (fig. S2B) of the spinal cord of mSOD1 mice revealed that anti-RGMa treatment demonstrated no effect on astrogliosis, microgliosis, or neuroinflammation. Immunohistochemical analysis of gastrocnemius muscles revealed that anti-RGMa antibodies did not affect the number of innervated neuromuscular junctions (NMJs) in both groups (fig. S3, A and B), suggesting that anti-RGMa antibody treatment had no positive impact on the NMJs of mSOD1 mice.

Thus, these observations suggest that anti-RGMa antibody therapy effectively inhibited neurodegenerative processes in animal models of ALS and possesses therapeutic potential for treating patients with ALS by reducing SOD1 and ubiquitin aggregation.

### Anti-RGMa antibody promoted cofilin phosphorylation and actin polymerization in mSOD1 mice and primary neuronal cultures

**Fig. 5.**
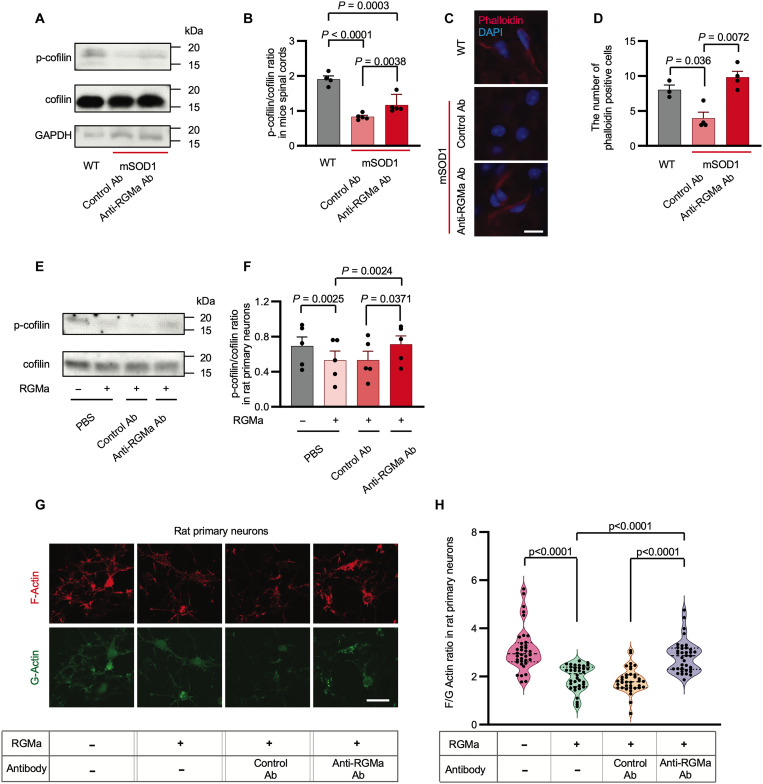
RGMa promoted cofilin dephosphorylation and actin depolymerization of mSOD1 mice and rat primary cortical neurons. (**A**) Immunoblotting assay of cofilin and p-cofilin in the spinal cord lysates of WT (*n* = 4) or mSOD1 mice treated with anti-RGMa (*n* = 5) or control Ab (*n* = 5). (**B**) P-cofilin/cofilin ratio in (A) is shown. (**C**) Immunohistochemical analysis of the spinal cord of WT (*n* = 3) and mSOD1 mice treated with anti-RGMa (*n* = 4) or control Ab (*n* = 4) is shown. Slices were stained with phalloidin (red) to reveal actin filaments and DAPI (blue) to reveal the cell nuclei. Scale bar, 30 μm. Representative images are shown. (**D**) There are a smaller number of phalloidin-positive motor neurons in mSOD1 mice than in WT controls. (**E**) Immunoblots of cofilin and p-cofilin in rat primary neurons lysates with or without recombinant RGMa, treated with anti-RGMa or control Ab. (**F**) Quantification of the relative intensity of p-cofilin/cofilin bands in (E). Data from three independent blots were used for the statistics. (**G**) Immunocytochemical analysis of rat primary neurons with or without RGMa, inoculated with anti-RGMa or control Ab were stained with phalloidin (red) and DNaseI (green) to reveal F-actin and G-actin, respectively. Representative images are shown. Scale bar, 30 μm. (**H**) Quantifications of F/G ratio are shown. Each filled dot indicates the ratio of F-actin mean intensity to G-actin mean intensity in a single cell. In all the conditions, a total of 36 cells selected from three individual wells of 16-chamber slides were measured (data are representative of two individual experiments). Error bars indicate mean ± SEM. p-cofilin, phosphorylated cofilin; F-actin, filamentous actin; G-actin, globular actin.

On the basis of previous reports in which RGMa signals regulate small GTPases, such as Rho, Rac, and Ras (*2*), resulting in the alteration of actin dynamics (*8*, *9*, *32*), and abnormal actin kinetics could be a starting point for ALS pathogenesis (*30*, *33*), we tested whether the positive effect of the anti-RGMa antibody observed in the present animal study could be attributed to the modulation of actin dynamics. Cofilin plays a critical role in depolymerizing filamentous actin (F-actin) at the slow-growing ends, creating new actin monomers (*34*). As cofilin is inactivated by phosphorylation, we examined the phosphorylation status of cofilin in the spinal cord of mSOD1 mice with or without anti-RGMa antibody treatment. Western blotting analysis revealed cofilin dephosphorylation to be significantly elevated in the spinal cord of mSOD1 mice, which was inhibited by the anti-RGMa antibody (Fig. 5, A and B). In addition, Western blotting analysis revealed that the amount of profilin in spinal cords did not change among WT mice, mSOD1 mice treated with anti-RGMa antibodies, and mSOD1 mice treated with control antibodies (fig. S4, A and F). Consistent with these observations, F-actin staining revealed that actin depolymerization was increased in the spinal cord of mSOD1 mice, which was cancelled by the anti-RGMa antibody (Fig. 5, C and D). In addition, we investigated the activation of Smad and NEO1 cleavage, which has been reported as a pathway excluding actin dynamics stimulated by RGMa/NEO1 signaling. Western blotting analysis demonstrated that Smad1, Smad2, and Smad3 phosphorylation did not change among WT and mSOD1 mice treated with anti-RGMa and control antibodies (fig. S4, B to D). NEO1 cleavage, which was induced upstream of the LIM domain only 4 (LMO4) activation (*35*) and regulates apoptosis induction, was not observed in either WT or mSOD1 mice (fig. S4E). The Western blot quantification is presented in fig. S4 (G to J). These data support our hypothesis that anti-RGMa therapy halts neurodegeneration in ALS by reinforcing actin polymerization.

Consistent with our observations in the spinal cord of mSOD1 mice, in vitro analysis revealed that recombinant soluble RGMa (rRGMa) reduced the phosphorylation of cofilin in rat primary neurons and that anti-RGMa antibody cancelled this effect (Fig. 5, E and F). Phalloidin and deoxyribonuclease (DNase) I staining was performed to visualize F-actin and globular actin (G-actin), respectively. We found that rRGMa decreased the F-actin/G-actin ratio (F/G ratio), and anti-RGMa antibody recovered the F/G ratio (Fig. 5, G and H).

Collectively, these data demonstrated that the anti-RGMa antibody enhanced actin polymerization through cofilin phosphorylation, which could be associated with improved motor performance and extended survival of mSOD1 mice.

### Recombinant RGMa enhanced the entry of recombinant SOD1 protein into rat primary cortical neurons via actin depolymerization or actin barrier collapse

**Fig. 6.**
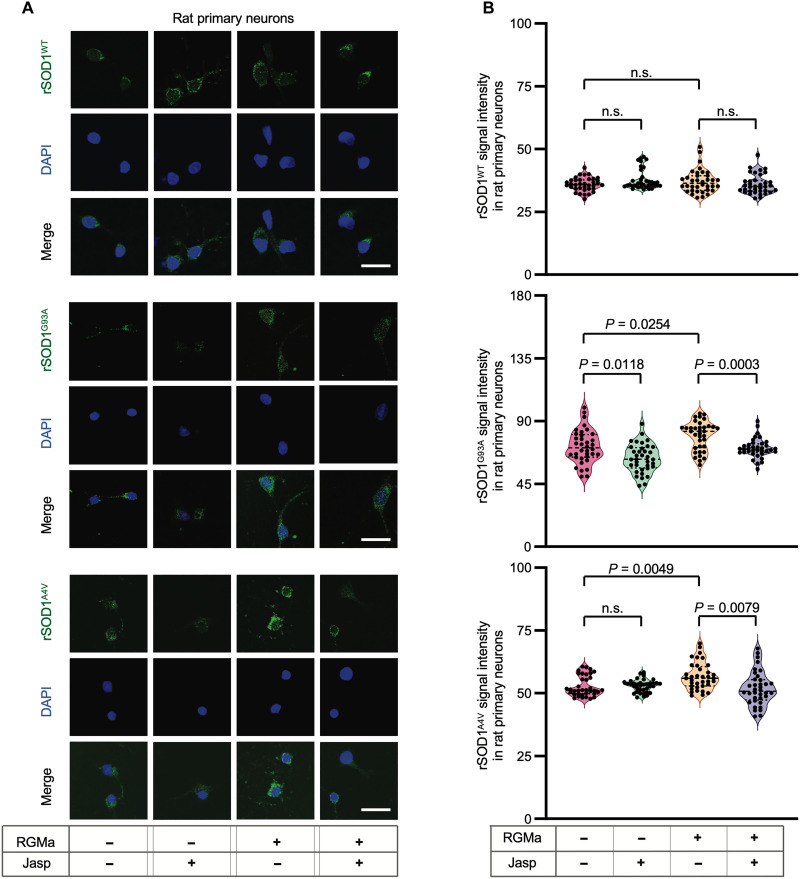
RGMa enhanced entry of mutant SOD1 protein into rat primary cortical neurons via F-actin reduction in vitro. RGMa and jasplakinolide were added to the rat primary neuron culture to evaluate the entry of rSOD1 proteins into the neurons. (**A**) Recombinant human WT SOD1 protein (rSOD1^WT^), recombinant human SOD1 protein with G93A mutation (rSOD1^G93A^), or one with A4V mutation (rSOD1^A4V^) labeled with FLAG was administered to rat primary neurons treated with or without RGMa and inoculated with jasplakinolide or DMSO. The rSOD1 protein was visualized using an anti-FLAG antibody conjugated to FITC. Representative images are shown. (**B**) Quantification of mean FITC intensity in rat primary neurons is shown in (A). Each filled dot indicates the mean FITC intensity of a single FITC-positive cell. In all the conditions, 36 cells selected from three individual wells were measured (Data are representative of two individual experiments). Scale bars, 20 μm. Error bars indicate the mean ± SEM.

Several lines of evidence suggest that the aggregation of mutant SOD1 species could be associated with the main driving mechanisms for ALS progression (*22*, *36*). The removal of mutant SOD1 protein through anti-SOD1 antibody treatment alleviated neurodegeneration in mSOD1 mice (*37*), highlighting the pathological involvement of extracellular proteins and their therapeutic potential. In addition, other studies described that F-actin, or the actin barrier, plays an important role in cell-to-cell transfer of mutant SOD1 protein aggregates (*33*). On the basis of these previous studies and our present results, we hypothesized that elevated variant C of RGMa in patients and animals with ALS accelerates the uptake of extracellular molecules by increasing “F-actin to G-actin conversion,” resulting in actin depolymerization and actin barrier collapse. To test this hypothesis, we first evaluated the uptake of recombinant soluble SOD1 (rSOD1) WT (rSOD1^WT^) and G93A (rSOD1^G93A^) or A4V (rSOD1^A4V^) mutations in the primary neurons of rats in vitro. Although rRGMa treatment did not increase the uptake of SOD1^WT^, it enhanced the uptake of mutant SOD1 by neurons, which was reversed by jasplakinolide, a stabilizing agent for actin polymerization (Fig. 6, A and B).

### Recombinant RGMa enhanced the entry of various molecules into rat primary cortical neurons via RGMa/NEO1 axis

**Fig. 7.**
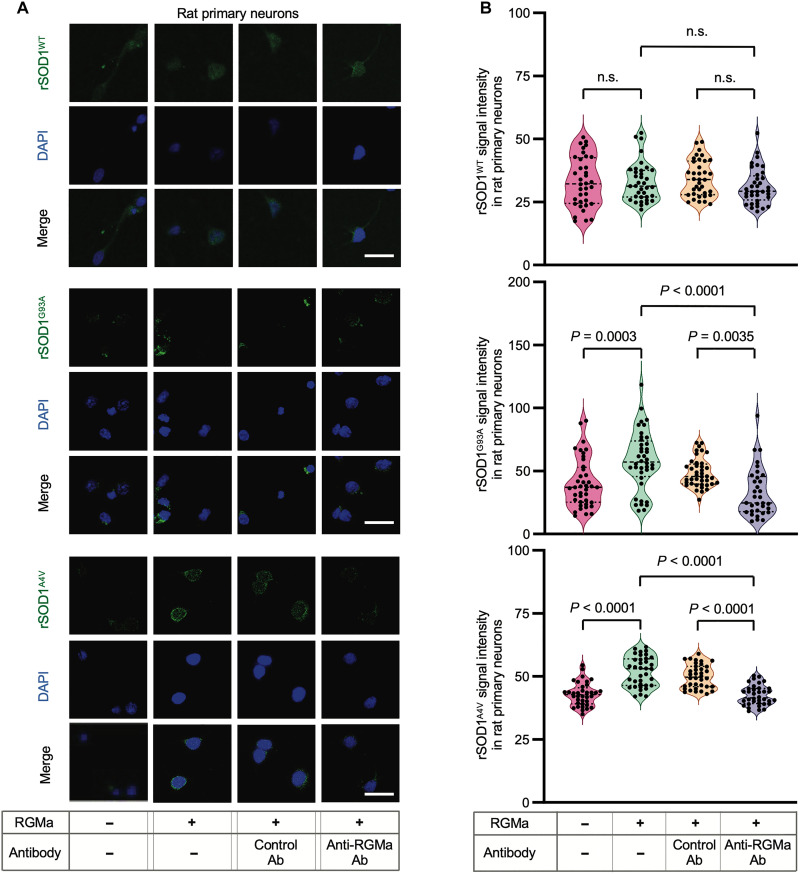
Anti-RGMa antibody suppressed entry of mutant SOD1 protein into rat primary cortical neurons in vitro. RGMa and anti-RGMa antibodies were added to rat primary neuron cultures to evaluate the entry of rSOD1 proteins into neurons. (**A**) rSOD1^WT^, rSOD1^G93A^, and rSOD1^A4V^ labeled with FLAG were administered to rat primary neurons treated with or without RGMa and inoculated with anti-RGMa Ab or control Ab. SOD1 protein in these cells was visualized using an anti-FLAG antibody conjugated with FITC. Representative images are shown. (**B**) Quantification of mean FITC signal intensity in rat primary neurons is shown in (A). Each filled dot indicates the mean FITC intensity of a single FITC-positive cell. Under all the conditions, 36 cells selected from three individual wells were measured (data are representative of two individual experiments). Scale bars, 20 μm. Error bars indicate the mean ± SEM.

To investigate the role of the RGMa/NEO1 axis on the uptake of extracellular molecules, we inhibited the effect of rRGMa by adding a monoclonal antibody. In vitro analysis revealed that anti-RGMa antibody canceled the effect of rRGMa on enhancing the uptake of extracellular rSOD1 protein (Fig. 7, A and B). In addition, we performed gene silencing of NEO1 in cultured primary neurons (fig. S5, A and B) and observed that the genetic knockdown of NEO1 canceled the effect of rRGMa on enhancing the uptake of extracellular rSOD1 protein (fig. S5, C and D).

To further investigate whether this observation is specific to rSOD1 or more relevant to other proteins, we evaluated the role of RGMa/NEO1 axis on recombinant human Tau protein (rTau), a representative protein that exhibits pathogenicity through aggregation (*22*, *28*). Cellular uptake of rTau by primary neurons was promoted by RGMa, but this effect was canceled by the anti-RGMa antibody (fig. S6, A and B) or genetic NEO1 silencing (fig. S6, C and D).

Overall, these findings suggest that RGMa in both patients and animals with ALS dephosphorylates cofilin via the RGMa/NEO1 axis, enhances actin depolymerization, resulting in the collapse of the actin barrier, and has a pivotal role in ALS pathogenesis by increasing mutant SOD1 uptake (fig. S7).

## DISCUSSION

While RGMa has previously been shown to be involved in the pathogenesis of neurological diseases, such as SCI (*11*, *12*), MS (*3*, *13*, *14*), and PD (*15*, *16*) through neuronal cell death, axonal damage, and inflammation promotion, the present study suggests that RGMa/NEO1 signaling is also altered in the spinal cord of patients with ALS and in animal models. Anti-RGMa neutralizing antibody administration in ALS animals exerted a neuroprotective effect, resulting in a decrease in the SOD1-positive aggregates in motor neurons and a significant prolongation of survival in ALS animal models. Notably, the in vitro analysis revealed that RGMa disrupted the actin barrier and increased the uptake of extracellular mutant SOD1 proteins by dephosphorylating cofilin and decreasing the F/G ratio. We found that the anti-RGMa antibody could exert its effects by reducing the propagation of pathogenic proteins (fig. S6).

We found that RGMa with its C-terminal domain (variant C) was increased in the CSF of patients with ALS than in those with ONDs, such as MS and PD, and in NDs such as normal pressure hydrocephalus and muscle disease, raising the possibility that the RGMa/NEO1 axis is more essentially involved in the pathogenesis of ALS than in other diseases. Within the CNS in ALS, spatial diffusion of soluble variant C of RGMa and altered localization of NEO1 resulted in downstream NEO1 signaling activation, which was not observed under physiological conditions, resulting in accelerated neurodegeneration in ALS. Recent reports have demonstrated RGMa to have a variety of neuropathic effects, including axon guidance (*2*), cell death induction (*5*), axonal damage promotion (*10*), inflammation induction (*3*, *4*), and angiogenesis induction (*7*), which may contribute to the ALS pathogenesis. The positive correlation between the concentration of RGMa variant C in the spinal fluid of patients with ALS and their clinical symptoms and the prolonged survival of ALS mice treated with the anti-RGMa antibody suggest that the anti-RGMa antibody inhibits the above-mentioned neuropathogenic effects.

Small GTPases, such as Rho, Rac, and Ras, which subsequently regulate actin dynamics, play important roles in the RGMa/NEO1 axis (*2*). Notably, increasing evidence suggests that disruption of the actin dynamics could be attributed to ALS. Profilin-1 (PFN1), an actin monomer–binding protein essential for the regulation of actin polymerization, has been identified as a causative gene of familial ALS (*38*). PFN1 mutations have been reported to result in not only decreased F-actin arrangement (*38*) but also altered stress granule dynamics (*39*) and TAR deoxyribonucleic acid–binding protein of 43 kDa (TDP-43) aggregation (*38*, *40*). Transgenic mice with mutated PFN1 not only recapitulate paralysis and motor neuron degeneration resembling ALS (*41*) but also demonstrate TDP-43 aggregation pathology, suggesting that actin dynamics is intrinsically involved in ALS pathogenesis. Cofilin, which is essential for actin depolymerization, has also been associated with ALS pathology. A previous report demonstrated that cofilin is associated with the reduction of F-actin in induced pluripotent stem cell–derived motor neurons in patients with ALS with GGGGCC intronic repeat expansion in *C9orf72*, a common genetic form of familial ALS (*42*). In addition, cofilin dephosphorylation results in actin disassembly and impairs the actin barrier to exploit the entry of protein aggregates (*33*). We have demonstrated that phosphorylated cofilin and F-actin are decreased in primary rat neurons upon addition of rRGMa, which is restored by an anti-RGMa antibody. Consistent with these findings, both patients and mice with ALS showed increased CSF RGMa level and decreased phosphorylated cofilin and F-actin levels in the spinal cords of ALS mice, which were restored by anti-RGMa antibody administration. This suggests that RGMa, which increases in the CSFs of both patients and mice with ALS, may accelerate neurodegeneration in ALS by inducing dephosphorylation of cofilin and depolymerization of F-actin.

Many previous studies have demonstrated that the cell-to-cell transmission of pathogenic proteins, such as SOD1 and TDP-43, drives the progression of ALS pathology, as shown in animal experiments in which these proteins were seeded (*22*, *23*). Some previous reports showed that the administration of anti-human SOD1 antibody improved disease symptoms, prolonged their lifespan, and reduced the aggregation of misfolded SOD1 protein and motor neuron degeneration in mSOD1 mice (*37*). These results suggest that removal of extracellular SOD1 induced the interference with the prion-like spreading of SOD1 aggregates released from dying cells or living cells. Endocytosis, modulated through actin dynamics, promoted the uptake of extracellular molecules, which served as the key mechanism of the propagation (*33*, *43*). Moreover, some previous reports demonstrated endocytosis to be modulated by axon guidance molecules, such as semaphorin 3A and EphrinA2, which alter actin dynamics (*44*, *45*). In this study, we demonstrated that cofilin-1 dephosphorylation and actin depolymerization by RGMa/NEO1 signaling promoted the cellular uptake of the SOD1 protein in vitro. This indicates that increased RGMa levels in ALS could disrupt the actin barrier and allow the entry of extracellular pathogenic proteins into the cell. In addition, we demonstrated that anti-RGMa antibody administration reduces SOD1 protein deposition in the spinal cord of ALS animals. This result could indicate that the anti-RGMa antibody not only ameliorates cell death but also restores the actin barrier disrupted by increased RGMa level, resulting in fewer mutant proteins entering the neurons.

Our study has certain limitations. First, we could not exclude the possibility that the anti-RGMa antibody exerts its effect not on neurons but on other CNS cells. Second, more studies are needed to reveal whether the effects of RGMa/NEO1 signaling, other than the transformation of F-actin to G-actin, are involved such as regulation of apoptosis. Third, we could not exclude the possibility of the alteration of the F/G ratio having a favorable effect on mSOD1 mice through mechanisms other than prevention of protein aggregation, such as nucleocytoplasmic transport (*46*). Fourth, further studies, including in vivo experiments, are required to confirm whether RGMa promotes pathogenic protein propagation. Fifth, to avoid any gender bias in our preclinical animal research on ALS, we opted to only include female mSOD1 mice (*47*). However, we acknowledge that excluding male mice could also potentially introduce some bias in terms of clinical application. Sixth, we used only mSOD1 mice as animal models for ALS in our animal study, although we have several other options such as TDP-43, fused in sarcoma (FUS), or PFN1 transgenic mice.

This study demonstrated that the RMGa/NEO1 axis plays an important role in the collapse of the actin barrier to promote the propagation of pathogenic proteins in ALS, serving not only as a diagnostic and prognostic biomarker but as a potential therapeutic target. Further studies are warranted to determine whether RGMa-targeted therapies can improve other neurodegenerative diseases attributed to protein aggregation, including ALS.

## MATERIALS AND METHODS

### Ethics approval

All experiments were conducted in accordance with the revised Declaration of Helsinki and Good Clinical Practice guidelines and were approved by the ethics committee of Osaka University Hospital (permit number 12091-6, nos. 12148 and 20043). Written informed consent was obtained from all the participants or their legal representatives. All the animal experiments were performed in compliance with the Japanese national guidelines and the guidelines of the Osaka University (permit number: Biken-AP-H21-28-0).

### Patient information

A total of 30, 40, and 24 Japanese patients with ALS, ONDs, and NDs, respectively, who were hospitalized in the neurology department of Osaka University Hospital between November 2016 and March 2018 were enrolled in this study. All patients with ALS fulfilled the Awaji criteria for the diagnosis of ALS (definite, probable, or possible ALS) (*48*). We recruited 40 patients with ONDs (7 with MS, 5 with neuromyelitis optica, 14 with PD, 8 with dementia with Lewy bodies, 4 with chronic inflammatory demyelinating polyneuropathy, and 2 with spondylosis) and 24 patients with NDs (9 with normal pressure hydrocephalus, 13 with peripheral nerve or muscle disease, and 2 with functional neurological symptom disorder) (table S1). The demographic and clinical characteristics of patients with ALS were obtained from the medical records and directly from the patients. The progression rate of patients with ALS (ΔFS) until CSF analysis was calculated as follows: ΔFS = [48 − the revised ALS functional rating scale (ALSFRS-R) at the time of diagnosis]/duration from onset to diagnosis (months). These data are summarized in table S2. Autopsy samples were obtained from three patients with ALS and three control patients without neurodegenerative diseases (table S3).

### Animal studies and anti-RGMa antibody administration

All the mice were housed in microisolator cages within a modified pathogen-free barrier facility at the Animal Resource Center of Osaka University and provided food and water ad libitum. Transgenic mice overexpressing the familial ALS–associated G93A SOD1 mutation (harboring a single glycine to alanine substitution at codon 93) were obtained from the Jackson Laboratory (strain designated as B6SJL-TgN [SOD1- G93A]1Gurd/J) and backcrossed with C57BL/6 mice for at least 10 generations. We followed the guidelines (*47*) and we used only female mice for the experiments of survivals and clinical scores, to exclude the influence of gender, and we evaluated the relative copy number of mutant *superoxide dismutase 1* (*mSOD1*) using qPCR (*47*), confirming the absence of a significant difference in the copy number among the groups in our experiments.

The anti-RGMa monoclonal antibody and control antibody (palivizumab) were provided by the Mitsubishi Tanabe Pharma Corporation, Japan. Antibodies were intraperitoneally administered to 12 female SOD1 mice (250 μg twice per week). Antibody administration was started at 6 weeks of age and continued until the mSOD1 mice exhibited complete body paralysis. Body weight was measured three times per week as an indicator of muscle atrophy, which was indicated as the ratio of body weight at 10 weeks old. Motor function was evaluated using a rotarod apparatus (Rotarod treadmill for mice, MK-610A, Muromachi Kikai Co. Japan). The rotation of the rotarod cylinder was accelerated to 15 rpm in 120 s and revolved at a constant speed of 15 rpm for 300 s. Each mouse was subjected to three trials, and the longest latency without falling was recorded.

### CSF analysis

The CSF of patients were collected and immediately centrifuged at 400*g* for 10 min at 4°C. CSF supernatants were stored at −80°C until use. CSF was collected from 70-, 120-, and 150-day-old mSOD1 mice and WT control mice. The neck backs of the mice were excised under the combination of three anesthetics [medetomidine (0.3 mg/kg), midazolam (4.0 mg/kg), and butorphanol (5.0 mg/kg)], and their dura maters were punctured using a 30-gauge needle, as described previously (*49*). Ten microliters of CSF were collected from the cisterna magna under a binocular stereomicroscope. CSF was immediately placed on ice and briefly centrifuged to remove any red blood cells. Water (10 μl) was added to the pellet and hemoglobin released from the red blood cells in the pellet was detected through absorbance at 417 nm using a NanoDrop 2000 spectrophotometer (Thermo Fisher Scientific) to assess blood contamination in the CSF. Mouse CSFs without any blood contamination were stored at −80°C until use.

The RGMa concentration in the CSF of both patients and mice was measured using the ELISA kit [R&D systems, DY2459-05 (human) and MRGMA0 (mouse)] according to the manufacturer’s instructions. These ELISA recognized both the N terminus and C terminus of RGMa.

A sandwich ELISA using a combination of antigen and anti-human immunoglobulin G (IgG) antibody was established to quantify the anti-RGMa antibody in mouse CSF. A microplate was coated with 20 ng of recombinant human RGM-A protein (R&D Systems Inc.) per well and subsequently blocked with 1% bovine serum albumin. The wells were washed and incubated with 100 μl of diluted CSF sample. After the plate was washed, 5 ng of biotinylated goat anti-human IgG antibody (SouthernBiotech) was added to each well and incubated. The plate was washed and incubated with horseradish peroxidase (HRP)–conjugated streptavidin (ImmunoBioScience). Last, humanized anti-RGMa antibody was quantified using 1-Step Ultra TMB-ELISA Substrate Solution (Thermo Fisher Scientific), and absorbance at 450 nm was measured using an EnSpire 2300 Multilabel Reader (PerkinElmer Co. Ltd.).

### Co-immunoprecipitation/immunoblotting of human and mice CSF

Human CSF (100 μl) was added to the protease and phosphatase inhibitors and immunoprecipitated with 20 μl of protein G sepharose and 2 μg of anti-RGMa antibody (Santa Cruz Biotechnology; sc393046), which binds to amino acids 291 to 365 of human RGMa.

For the animal study, 5 μl of mouse CSF was added to 100 μl of phosphate buffer solution (PBS) with protease and phosphatase inhibitors immunoprecipitated with 20 μl of protein G sepharose and 0.88 μg of anti-RGMa antibody (Proteintech; 12387-1-ap), which binds to amino acids 136 to 485 in mice RGMa.

The samples were treated with 20 μl of sample buffer (Bio-Rad) and boiled at 95°C for 5 min. Boiled samples were resolved on SDS–polyacrylamide gel electrophoresis (SDS-PAGE) and transferred onto polyvinylidene difluoride (PVDF) membranes (ATTO). These blots were incubated at 4°C overnight with rabbit anti-RGMa antibody (1:500, Abcam; ab169761), which binds to amino acids 400 to 454 at the C terminus in the human analysis, or with rabbit anti-RGMa antibody (Proteintech; 12387-1-ap) in the animal analysis. They were subsequently incubated with the appropriate HRP-conjugated secondary antibodies for 60 min at room temperature and visualized using the ChemiDoc Touch Imaging System (Bio-Rad). The images were analyzed using ImageJ and image-processing software (www.imagej.net).

### Immunoblotting of mice CSF to measure extracellular SOD1 protein

Three microliters of mouse CSF (WT, *n* = 3; mSOD1 mice treated with anti-RGMa antibody, *n* = 3; and mSOD1 mice treated with control antibody, *n* = 3) was treated with 4 μl of sample buffer (Bio-Rad), 1.5 μl of 2-mercaptoethanol, and 7 μl of cold PBS and boiled at 95°C for 5 min. Seven microliters of boiled samples were resolved on SDS-PAGE and transferred onto PVDF membranes (ATTO). These blots were incubated at 4°C overnight with sheep anti-SOD1 antibody (1:4000, Sigma-Aldrich; 574597). They were subsequently incubated with the appropriate HRP-conjugated secondary anti-sheep antibodies (1:1000, R&D; HAF016) for 60 min at room temperature and visualized using the ChemiDoc Touch Imaging System (Bio-Rad). The images were analyzed using ImageJ and image-processing software (www.imagej.net).

### Histological examination of human autopsy samples

Formalin-fixed paraffin-embedded human lumbar cord (L5) tissues are shown in table S3. Three were cut at a thickness of 6 μm. Deparaffinized sections were incubated for 30 min with 0.3% H_2_O_2_ to quench endogenous peroxidase activity, washed with PBS three times, and blocked with blocking buffer of the VECTASTAIN ABC Kit (Vector Laboratories, PK-4002) for 30 min at room temperature. Sections were then incubated with mouse antibodies against NEO1 (1:100 dilution, Santa Cruz Biotechnology) overnight at 4°C. Autoclaving was performed for 15 min before incubation with the primary antibody. After rinsing with PBS three times, the sections were incubated with goat anti-mouse Igs conjugated to peroxidase-labeled dextran polymer (Dako Envision+, Dako) as a secondary antibody. Reaction products were visualized using 3,3′-diaminobenzidine tetrahydrochloride (Vector Laboratories, Burlingame, CA, USA). Hematoxylin was used to counterstain the cell nuclei. Images were obtained using an all-in-one fluorescence microscope BZ-X710 (Keyence, Osaka, Japan) with a 40× object.

### Histological examination of the mice spinal cord samples

Paraffin-embedded spinal cord sections of mSOD1 mice were prepared and subjected to immunohistochemistry, as previously described (*50*, *51*). In brief, the mSOD1 and control mice were euthanized at 105 days old and perfused with 4% paraformaldehyde (PFA). Lumbar spinal cords were dissected and dehydrated in increasing alcohol concentrations before embedding them in paraffin. Fifteen serial horizontal sections of the L5 segment of the spinal cord (3 μm thick) were prepared every 10 μm using a microtome and mounted onto slides. Deparaffinized sections were incubated for 30 min with 0.3% H_2_O_2_ to quench endogenous peroxidase activity and washed with PBS.

Nissl staining was performed on deparaffinized spinal cords of mSOD1 mice treated with control antibody (*n* = 5) or anti-RGMa antibody (*n* = 5) for the motor neuron count; the motor neurons in both the ventral horns on each section were counted, as previously described (*51*). Images were obtained using an all-in-one fluorescence microscope BZ-X710 (Keyence, Osaka, Japan) with a 40× object. Neurons containing somas with an area of >300 μm^2^ were considered motor neurons. All the experiments were conducted in a blinded manner.

For immunohistochemistry, deparaffinized spinal cords of mSOD1 mice were blocked with the blocking buffer of the VECTASTAIN ABC Kit (Vector Laboratories, PK-4001 for rabbit primary antibody or PK-4002 for mouse primary antibody) for 30 min at room temperature. The following primary antibodies were used: mouse NEO1 monoclonal antibody (1:100 dilution, Santa Cruz Biotechnology) (WT, *n* = 5 and mSOD1 mice, *n* = 5), rabbit polyclonal antibody against ubiquitin (1:1000 dilution, DAKO) (control antibody group, *n* = 3 and anti-RGMa antibody group, *n* = 7), and mouse monoclonal antibody against SOD1 (1:100 dilution, MBL) (control antibody group, *n* = 4 and anti-RGMa antibody group, *n* = 6). Autoclave treatment was performed for 15 min before incubation with all the antibodies. The following secondary antibodies were used: goat anti-mouse and anti-rabbit Igs conjugated to a peroxidase-labeled dextran polymer (Dako Envision+, Dako). Reaction products were visualized using 3,3′-diaminobenzidine tetrahydrochloride (Vector Laboratories, Burlingame, CA, USA). Hematoxylin was used to counterstain the cell nuclei. The ubiquitin- and SOD1-positive regions of interest (ROIs) included the complete anterior horn. ImageJ was used to quantify the percentage of the area positive for misfolded SOD1 immunoreactivity in the ROIs, as previously described (*37*).

Frozen spinal cord sections of mSOD1 mice treated with control antibody (*n* = 3) and anti-RGMa antibody (*n* = 3) were prepared and subjected to immunohistochemistry as previously described (*51*). The following primary antibodies were used: mouse glial fibrillary acidic protein (GFAP) monoclonal antibody conjugated with Alexa Flour 488 (1:50; Cell Signaling Technology) and rabbit anti-ionized calcium-binding adapter molecule 1 (Iba1) polyclonal antibody (1:500; Wako). Goat anti-rabbit IgG antibody conjugated to Alexa Flour 488 (1:200, Invitrogen) was used as the secondary antibody. After three washes with PBS, the samples were mounted with VECTASHIELD Mounting Medium containing 4′,6-diamidino-2-phenylindole (DAPI) (Vector Laboratories, H-1200). Images were obtained using an all-in-one fluorescence microscope BZ-X710 (Keyence, Osaka, Japan) with a 40× object.

### Histological examination of the mice muscle samples

Frozen gastrocnemius muscles of 100-day-old mSOD1 mice treated with control antibody (*n* = 3) or anti-RGMa antibody (*n* = 3) were cut at a thickness of 20 μm and subjected to immunohistochemistry as previously described (*52*–*54*). The following primary antibodies were used: mouse Synaptosomal-associated protein, 25 kDa (SNAP25) monoclonal antibody (1:5000, BioLegend: SM1-81) and α-bungarotoxin conjugated with Alexa Flour 488 (1:100; Thermo Fisher Scientific: B13422) to visualize the presynaptic terminals and postsynaptic nicotinic acetylcholine receptor (AChR), respectively. Goat anti-mouse IgG antibody conjugated to Alexa Flour 594 (1:200, Invitrogen: A-11005) was used as the secondary antibody. After three washes with PBS, the samples were mounted with VECTASHIELD Mounting Medium containing DAPI (Vector Laboratories, H-1200). Images were obtained using confocal microscopy FV3000 (Olympus) at 20× magnification. To determine the percentage of innervated NMJs, we used this formula: % = (number of AchR merged with SNAP25 signals/total number of AchR).

### RNA extraction and real-time qPCR analysis of the spinal cords of the mice

We perfused male SOD1 (*n* = 3) and WT (*n* = 3) mice at 105 days each with cold PBS to generate frozen samples of the spinal cord as described previously (*50*, *51*). The entire spinal cord was cut out and stored at −80°C. Total RNA was prepared using ISOGEN2 (NIPPON GENE) and was used to generate complementary DNAs (cDNAs) with the Super Script VILO cDNA Synthesis Kit (Thermo Fisher Scientific) according to the manufacturer’s instructions. Thereafter, 1 μl of primer mix of the target gene and 10 μl of TaqMan universal PCR master Mix (Thermo Fisher Scientific) were added to dilute cDNA to 20 μl. RGMa gene expression was compared to that of β-actin using an optimal primer probe assay.

Similarly, we generated total RNA from the spinal cords of mSOD1 male mice at 100 days old treated with anti-RGMa antibody (*n* = 5) or control antibody (*n* = 5) and prepared RNA samples for TaqMan qPCR assay. The expression of the target genes, *ionized calcium-binding adapter molecule 1* (*Iba1*), *GFAP*, *interferon gamma* (*IFN-g*), and *nitric oxide synthase 2* (*NOS2*), was compared with β-actin using an optimal primer probe assay.

We used the following thermal cycle protocol for all sample assays: 2 min at 50°C and 10 min at 95°C, followed by 40 cycles of 15 s at 95°C and 1 min at 60°C using an Applied Biosystems 7900HT (Carlsbad, CA). The cycle threshold (CT) values were analyzed using SDS 2.4.1 software (Applied Biosystems).

### Phalloidin visualization of WT and mSOD1 mice with anti-RGMa or control antibody

Frozen spinal cord sections of WT (*n* = 3) mSOD1 mice treated with anti-RGMa antibody (*n* = 4) or control antibody (*n* = 4) were fixed with 4% PFA and exposed to 0.5% Triton X-100 in PBS for 15 min. After three washes with PBS, phalloidin conjugated with rhodamine (cytoskeleton, #PHDR1) (1:100) was applied overnight at 4°C. The samples were mounted with VECTASHIELD mounting medium containing DAPI (Vector Laboratories, H-1200). The number of rhodamine-positive cells, which were grossly detected in both the ventral horns, was counted using a microscope (BZ-x810, Keyence) with a 40× magnification.

### Western blotting of WT and mSOD1 mice with anti-RGMa or control antibody

Western blotting was performed as previously described (*51*). In brief, mSOD1 and WT mice (105 days old) were perfused with cold PBS. The whole spinal cord was dissected, homogenized on ice, and stored at −80°C. These samples were lysed with radioimmunoprecipitation buffer (PBS, 0.1% Triton X-100, 0.5% sodium deoxycholate, 0.1% SDS, 50 mM tris-HCl, and 150 mM NaCl, pH 8.0) containing protease and phosphatase inhibitors. Equal concentrations of proteins were resolved on 10% SDS-PAGE and then transferred onto PVDF membranes (ATTO, Tokyo, Japan). The blots were incubated at 4°C overnight with one of the following primary antibodies: anti-RGMa antibody (1:500, Abcam; ab26287), anti–β-actin antibody (1:50,000; Sigma-Aldrich), anti-cofilin antibody (1:80,000, Proteintech; 66057-1-Ig), anti–phospho-cofilin antibody (1:1000, Cell Signaling Technology; #3313), anti-neogenin antibody (1:2000, LifeSpan BioSciences; LS-B13131), anti-Smad1 antibody (1:100, Santa Cruz Biotechnology; sc-7965), anti-phospho-Smad1/5/9 (1:1000, Cell Signaling Technology; #13820), anti-Smad2/3 antibody (1:1000, Cell Signaling Technology; #3102), anti–phospho-Smad2/3 antibody (1:1000; Cell Signaling Technology; #8828), anti-profilin antibody (1:100, Santa Cruz Biotechnology; sc137236), and anti–glyceraldehyde-3-phosphate dehydrogenase (GAPDH) antibody (1:1000; Cell Signaling Technology; M171-3). The blots were subsequently incubated with the appropriate HRP-conjugated secondary antibodies for 90 min and visualized using SuperSignal West Femto Maximum Sensitivity Substrate (Thermo Fisher Scientific, Waltham, MA, USA). The image of each band was captured using Image Gauge (Fuji Film, Japan) and analyzed using ImageJ software.

### Primary culture of rat cortical neurons

Primary cultures of rat cortical neurons were obtained as described previously (*55*). In brief, neuronal cultures were prepared from the cortex at embryonic day 16 (E16). The cortices were dissociated using a papain dissociation system (LK003150; Worthington). These cells were harvested in 24-transwell plates, 12-transwell plates, 16-chamber, or 8-chamber slides coated with poly-l-lysine (Sigma-Aldrich, P1274-25MG) and cultured in high-glucose Dulbecco’s modified eagle medium containing 5% fetal bovine serum and penicillin (100 IU/ml) at 37°C in a humidified atmosphere of 95% air and 5% CO_2_. After 24 hours, the medium was changed to the differentiation medium [Neuro Stem Cell Basal Medium (Merck, SCM003) supplemented with B-27 (Invitrogen, 12587010)], and half of the medium change was performed every 2 to 3 days for 7 to 8 days.

Primary neurons were seeded on 16-chamber slides (10,000 cells per well). After 7 days of culture, the cells were treated with recombinant human RGMa (rRGMa) (2 μg/ml) (NovoPro, 500040) or deionized distilled water (DDW) and anti-RGMa antibody or control antibody (20 μg/ml). After 30 min of incubation, the primary neurons were fixed with 4% PFA or methanol and acetone and used in the subsequent analyses.

### Evaluation of the F/G ratio

Primary neurons were seeded on 16-chamber slides (10,000 cells per well). After 7 days of culture, the cells were treated with rRGMa (2 μg/ml) (NovoPro, 500040) or DDW and anti-RGMa antibody or control antibody (20 μg/ml). After 30 min of incubation, the primary neurons were fixed with methanol and acetone at −20°C for 20 min. F-actin in the primary neurons was labeled with phalloidin conjugated with rhodamine for 40 min. These primary neurons were subsequently treated with 0.1% Triton X-100 for 5 min before the G-actin in them was stained with DNase I conjugated with Alexa Fluor 488 (Molecular Probes, D12371) for 20 min. The F/G ratio was determined by comparing the mean intensity of rhodamine and Alexa Fluor 488 in 36 neurons from three individual wells using ImageJ. The samples were mounted with VECTASHIELD mounting medium containing DAPI (Vector Laboratories, H-1200). Images were obtained using a confocal microscope ZEISS LSM 880 with Airyscan (AZ Science) with 40× magnification.

### Generation of recombinant SOD1 protein

The expression vectors of human rSOD1^WT^ and mutated with G93A (rSOD1^G93A^) and A4V (rSOD1^A4V^) were provided as previously described (*56*). These SOD1-FLAG plasmids encoded a fusion protein consisting of SOD1 at the N terminus, a linker sequence of GSRA, and a triplet FLAG epitope tag at the C terminus. Human embryonic kidney cells (HEK) 293T cells were seeded at 7 × 10^6^ cells per dish in 100 × 20–mm cell culture dishes and transfected with 10 μg of the vectors carrying mutated SOD1-FLAG using FuGENE6 transfection reagent (Promega, E2961). Forty-eight hours after transfection, the cultured cells were homogenized in 500 μl of buffer B (50 mM tris and 150 mM NaCl, pH 7.4). The extracts of HEK293T cells (500 μl) were incubated with 50 μl of anti-FLAG M2-agarose affinity gel (Merk, A2220-1ML) at 4°C overnight. The resin was washed three times with buffer B. SOD1-FLAG proteins were eluted from the resin by competition with the 3× FLAG peptide (Protein Ark, F4799-4MG) for 1 hour. Last, we measured the mutated SOD1 concentration using ELISA (Abcam, ab119520).

### Visualization of intracellular recombinant SOD1 protein

Rat primary neurons were prepared on 16-chamber slides (10,000 cells per well). After 7 days of culture, the cells were treated with rRGMa (2 μg/ml) or DDW, and jasplakinolide (250 nM) and dimethyl sulfoxide (DMSO). Primary neuron cultures were incubated with rSOD1 protein (20 nM) for 60 min and fixed with methanol and acetone at −20°C for 20 min to evaluate SOD1 entry into the neurons. Cells were labeled with a mouse monoclonal anti-FLAG M2 antibody conjugated with fluorescein isothiocyanate (FITC) (Sigma-Aldrich, F4049, 1:100) overnight at 4°C. We performed similar experiments by substituting the anti-RGMa antibody and control antibody (20 μg/ml) for jasplakinolide and DMSO to investigate the effect of jasplakinolide on rSOD1.

These samples were mounted using VECTASHIELD mounting medium containing DAPI (Vector Laboratories, H-1200). Images were obtained using a confocal microscopy FV3000 (Olympus) at 40× magnification. We evaluated the mean intensity of FITC in 36 neurons from three individual wells using ImageJ software.

### Visualization of intracellular Tau protein

Rat primary neurons, cultured for 7 days, were treated with rRGMa (2 μg/ml) or DDW and, at the same time, with the anti-RGMa antibody or control antibody. After these primary neurons were incubated with 250 nM recombinant human Tau protein (AnaSpec, AS-55556-50) for 60 min, they were fixed with methanol and acetone at −20°C for 20 min. Cells were labeled with a mouse monoclonal anti–phospho-tau antibody (Invitrogen, MN1020, 1:100) overnight at 4°C and were subsequently stained with Alexa Fluor 594.

These samples were mounted using VECTASHIELD mounting medium containing DAPI (Vector Laboratories, H-1200). Images were obtained using a confocal microscopy FV3000 (Olympus) at 40× magnification. We evaluated the mean intensity of Alexa Fluor 594 in 36 neurons from two individual wells using ImageJ software.

### Visualization of intracellular rSOD1 and Tau protein with NEO1 knockdown

Primary neurons were prepared on eight-chamber slides (80,000 cells per well). After 24 hours of seeding, rat NEO1 small interfering RNA (siRNA) (Dharmacon, #L-101073-02-0005) and control siRNA (Dharmacon, #D-001810-10-05) (50 nM) were transfected with lipofectamine RNAiMax (Thermo Fisher Scientific, #313778–075) and reacted for 6 hours. After 5 days of incubation, the cells were treated with rRGMa (2 μg/ml) or DDW and incubated with rSOD1 protein (20 nM) or with rTau protein (250 nM) for 60 min and fixed with methanol and acetone at −20°C for 20 min. Anti-FLAG M2 antibody labeling with FITC and anti–phospho-Tau antibody conjugated with Alexa Fluor 594 was administered to stain rSOD1 and rTau respectively, as described above.

Images were obtained using a confocal microscopy FV3000 (Olympus) at 40× magnification. We evaluated the mean intensity of FITC or Alexa Fluor 594 in 36 neurons from two individual wells using ImageJ software.

For confirmation of NEO1 gene silencing, total RNA of rat primary cells was prepared using ISOGEN2 (NIPPON GENE) and was used to generate cDNAs with Superscript IV VILO Master Mix (Thermo Fisher Scientific, #11756050). Thereafter, 1 μl of primer mix of the target gene and 10 μl of TaqMan universal PCR master Mix (Thermo Fisher Scientific) were added to dilute cDNA to 20 μl. NEO1 gene expression was compared to that of GAPDH using an optimal primer probe assay (NEO1; Rn01417716_m1, GAPDH; Rn01775763_g1, Thermo Fisher Scientific). We used the following thermal cycle protocol for all sample assays: 2 min at 50°C and 10 min at 95°C, followed by 40 cycles of 15 s at 95°C and 1 min at 60°C using Quant Studio 7. The CT values were analyzed using SDS 2.4.1 software (Applied Biosystems).

To confirm the knockdown of NEO1, rat primary cells were lysed with radioimmunoprecipitation buffer (PBS, 0.1% TritonX-100, 0.5% sodium deoxycholate, 0.1% SDS, 50 mM tris-HCl, and 150 mM NaCl, pH 8.0) containing protease and phosphatase inhibitors. Equal concentrations of proteins were resolved on 5 to 20% SDS-PAGE and then transferred onto PVDF membranes (ATTO, Tokyo, Japan).

The blots were incubated at 4°C overnight with one of the following primary antibodies: anti-NEO1 antibody (1:500, Biorbyt; #orb499887), and anti–β-actin antibody (1:5000; Sigma-Aldrich; #A5441). The blots were subsequently incubated with the appropriate HRP-conjugated secondary antibodies for 30 min and visualized using Amersham ECL Prime Western Blotting Detection Reagent (Cytiva, #RPN2232). The image of each band was captured using Chemi Doc Touch (BioRad).

### Statistical analysis

For continuous data, Student’s *t* test or Mann-Whitney *U* test was used to compare the two groups. One-way analysis of variance (ANOVA) was used to compare more than three groups, and Tukey’s multiple comparisons test was subsequently performed to compare each group. The effect of RGMa concentration on ALS, NDs, and ONDs was analyzed using analysis of covariance to adjust for a between-group imbalance of age and sex (Fig. 1A). The ROC curve and the area under the curve (AUC) index were used for performance diagnosis to classify the types of diseases based on the RGMa level (Fig. 1B); Pearson’s correlation was used as an association measure between the two continuous datasets (Fig. 1D). Two-way repeated measures ANOVA was used to analyze multiple measures of the same variable assessed in the same subjects at two or more time periods (Figs. 3A and 4, B and C), and the generalized Wilcoxon test was used to compare the survival data between the two groups (Fig. 4A). Data are expressed as mean ± SEM or SD. Statistical significance was set at a *P* value of <0.05.
