# Supplementary material for: RGMa collapses the neuronal actin barrier against disease-implicated protein and exacerbates ALS
Source: Sci Adv. 2023 Nov 22;9(47):eadg3193. doi: 10.1126/sciadv.adg3193 (PMC10665002; doi:10.1126/sciadv.adg3193)
Supplement: Supplementary file 1 — Figs. S1 to S7 Tables S1 to S3 [file sciadv.adg3193_sm.pdf]

Science Advances
AAAS

Supplementary Materials for

RGMa collapses the neuronal actin barrier against disease-implicated protein and exacerbates ALS

Mikito Shimizu *et al.*

Corresponding author: Tatsusada Okuno, okuno@neurol.med.osaka-u.ac.jp;
Toshihide Yamashita, yamashita@molneu.med.osaka-u.ac.jp

Sci. Adv. **9**, eadg3193 (2023)
DOI: 10.1126/sciadv.adg3193

This PDF file includes:

Figs. S1 to S7
Tables S1 to S3

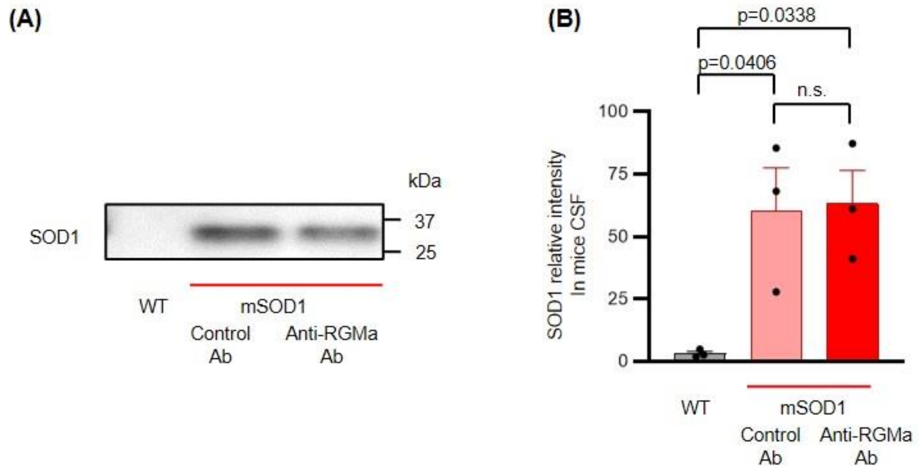

Fig. S1. Extracellular SOD1 protein was detected in the CSF of mSOD1 mice.
(A) Protein expression of mutant human SOD1 is upregulated in the CSF of mSOD1 mice compared to that in WT mice. Anti-RGMa antibody did not significantly affect the level of SOD1 protein expression in the CSF. **(B)** Relative intensity levels of the SOD1 protein are quantified in the CSF of each group of mice. Error bars indicate the mean ± SEM.
Ab, antibody; RGMa, repulsive guidance molecule A; mSOD1 mice, transgenic mice overexpressing the familial ALS-associated G93A SOD1 mutation; SEM, standard error of the mean

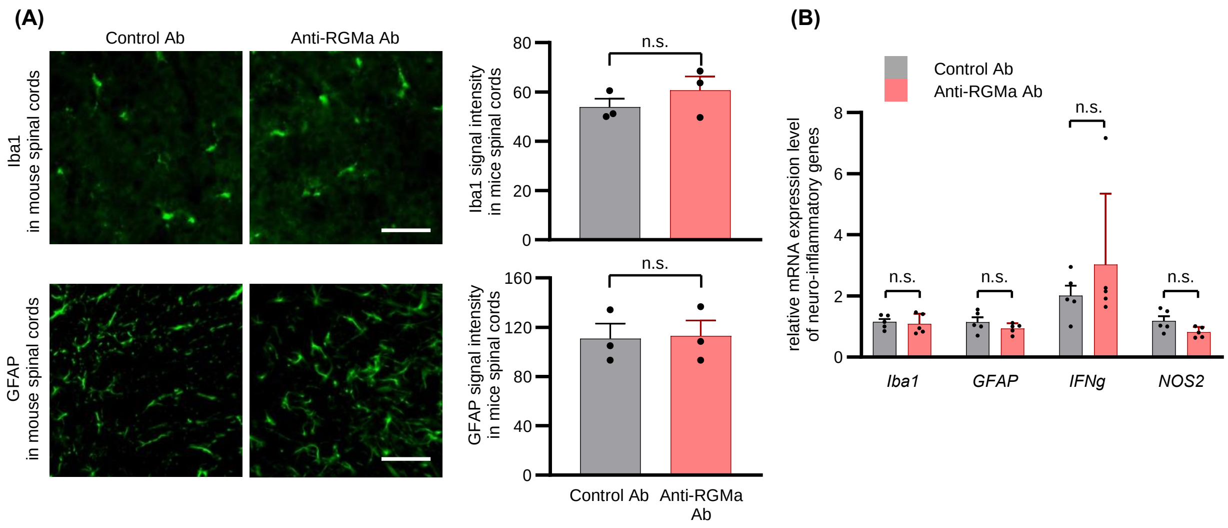

Fig. S2. Anti-RGMa antibody does not affect the activation of astrocytes and microglia in the mSOD1 mice.

(A) Immunofluorescent staining and quantitative analysis of the spinal cords of mSOD1 mice (105-days-old) treated with anti-RGMa Ab versus control Ab (n=3, each group). The samples of the mice's spinal cords were stained with anti-GFAP and anti-Iba1 antibodies; the representative images are shown above. Anti-RGMa Ab did not affect GFAP or Iba1 expression. Scale bar: 50 µm. **(B)** Quantitative PCR analysis revealed that anti-RGMa Ab treatment does not affect the relative expression levels of neuroinflammation-related genes (*Iba1*, *GFAP*, *IFN-g*, and *NOS2*) in the spinal cord of mSOD1 mice (100-days-old). Five animals from each group treated with the anti-RGMa and control Ab were used for this analysis. Error bars indicate the mean ± SEM.

Ab, antibody; RGMa, repulsive guidance molecule A; WT, wild-type; mSOD1 mice, transgenic mice overexpressing the familial ALS-associated G93A SOD1 mutation; GFAP, glial fibrillary acidic protein; Iba1, ionized calcium-binding adapter molecule 1; IFN-g, interferon gamma; NOS2, nitric oxide synthase 2; SEM, standard error of the mean

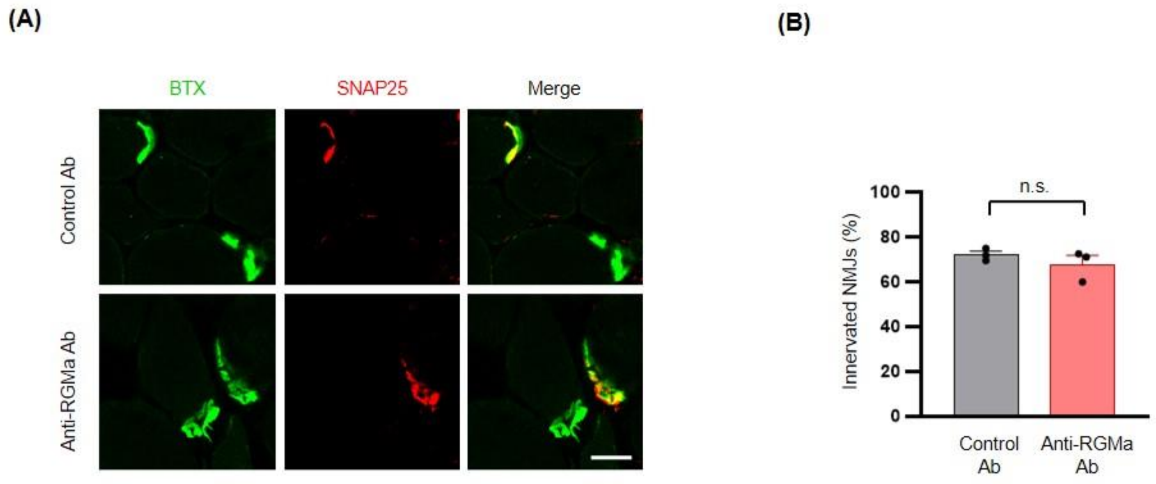

Fig. S3. Anti-RGMa antibody did not affect the integrity of neuromuscular junctions (NMJs) in mSOD1 mice.

(A) Representative immunofluorescence images of NMJs in the gastrocnemius of 100-days-old mSOD1 mice with anti-RGMa versus control Ab treatments (n=3 from each group). Samples were stained with Alexa Fluor 488 conjugated a-Bungarotoxin visualizing AchR, a marker of postsynaptic terminals (green) and anti-SNAP25 antibody visualizing presynaptic terminals (red). Co-localization of AchR with SNAP25 indicated innervated NMJs, while loss of SNAP25 demonstrated denervated NMJs. **(B)** Percentage of innervated NMJs in Figure S3A. Anti-RGMa antibody treatment had no positive impact on the NMJs of mSOD1 mice. Scale bar, 10 µm. Error bars indicate the mean ± SEM.

Ab, antibody; mSOD1 mice, transgenic mice overexpressing the familial ALS-associated G93A SOD1 mutation; RGMa, repulsive guidance molecule A; AchR, acetylcholine receptor; SNAP25, Synaptosomal-associated protein, 25kDa; NMJ, neuromuscular junctions. SEM, standard error of the mean

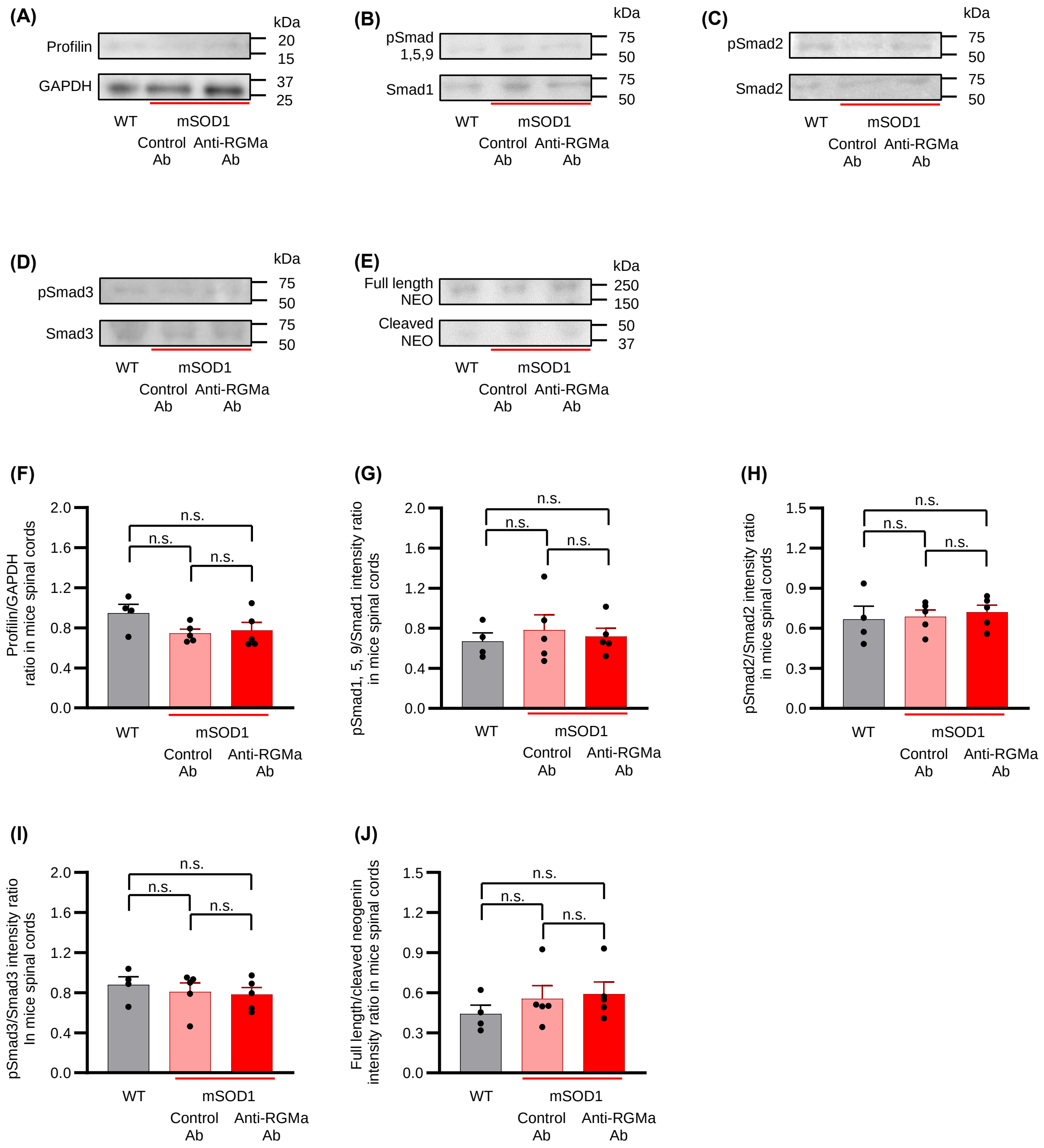

Fig. S4. Anti-RGMa antibody does not affect the expression level of profilin, the phosphorylation status of Smad or NEO1 cleavage in the spinal cord of the mSOD1 mice. Representative western blots showing **(A)** profilin and GAPDH, **(B)** p-Smad1, 5, 9/Smad1, **(C)** p-Smad2/Smad2, **(D)** p-Smad3/Smad3, and **(E)** full-length NEO1 and cleaved NEO1. **(F-J)** Relative expression levels of each molecule were quantified in three groups of 105-days-old mice: wild-type (n=4), mSOD1 mice treated with anti-RGMa Ab (n=5), and mSOD1 mice treated with the control antibody (n=5). Error bars indicate the mean ± SEM.

Ab, antibody; RGMa, repulsive guidance molecule A; NEO1, neogenin1; GAPDH, Glyceraldehyde-3-phosphate dehydrogenase; mSOD1 mice, transgenic mice overexpressing the familial ALS-associated G93A SOD1 mutation; SEM, standard error of the mean.

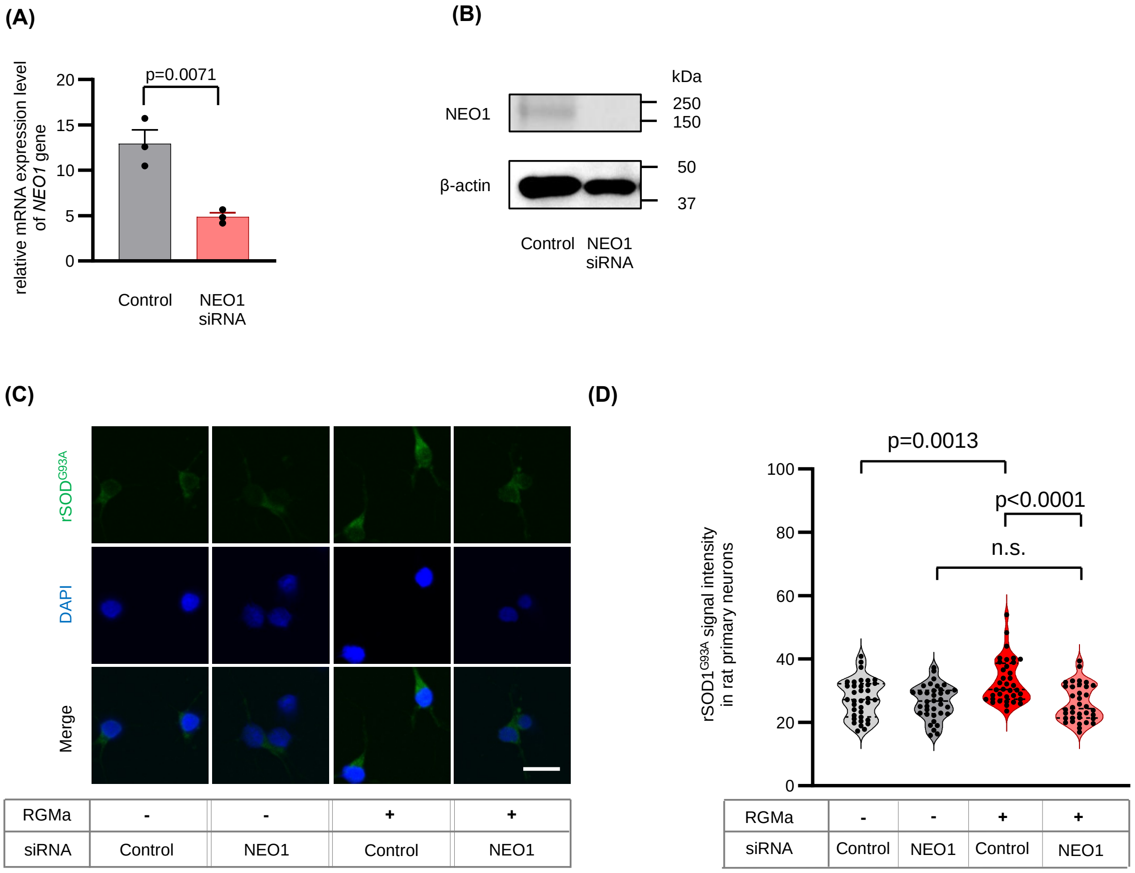

Fig. S5. Gene silencing of *NEO1* suppressed the entry of mutant rSOD1^{G93A} protein in rat primary cortical neurons in vitro.

(A) Quantitative PCR analysis confirmed that genetic silencing of *NEO1* reduced its transcript levels *in vitro*. **(B)** Western blots show that NEO1 knockdown suppressed the expression of NEO1 protein *in vitro*. **(C)** Rat primary neurons were treated with or without RGMa after gene silencing. Recombinant SOD1^{G93A} with FLAG-tag was administered thereafter and visualized using an anti-FLAG antibody. Representative immunofluorescence images are shown. **(D)** The mean fluorescence signal intensity in Figure S5C is quantified. Each filled dot indicates the mean fluorescence intensity of a single cell. Under all the conditions, 36 cells in 2 individual wells were selected. Error bars indicate the mean ± SEM.

NEO1, Neogenin 1; rSOD1^{G93A}, recombinant soluble superoxide dismutase 1 mutated with G93A; PCR, polymerase chain reaction; RGMa, repulsive guidance molecule A. SEM, standard error of the mean

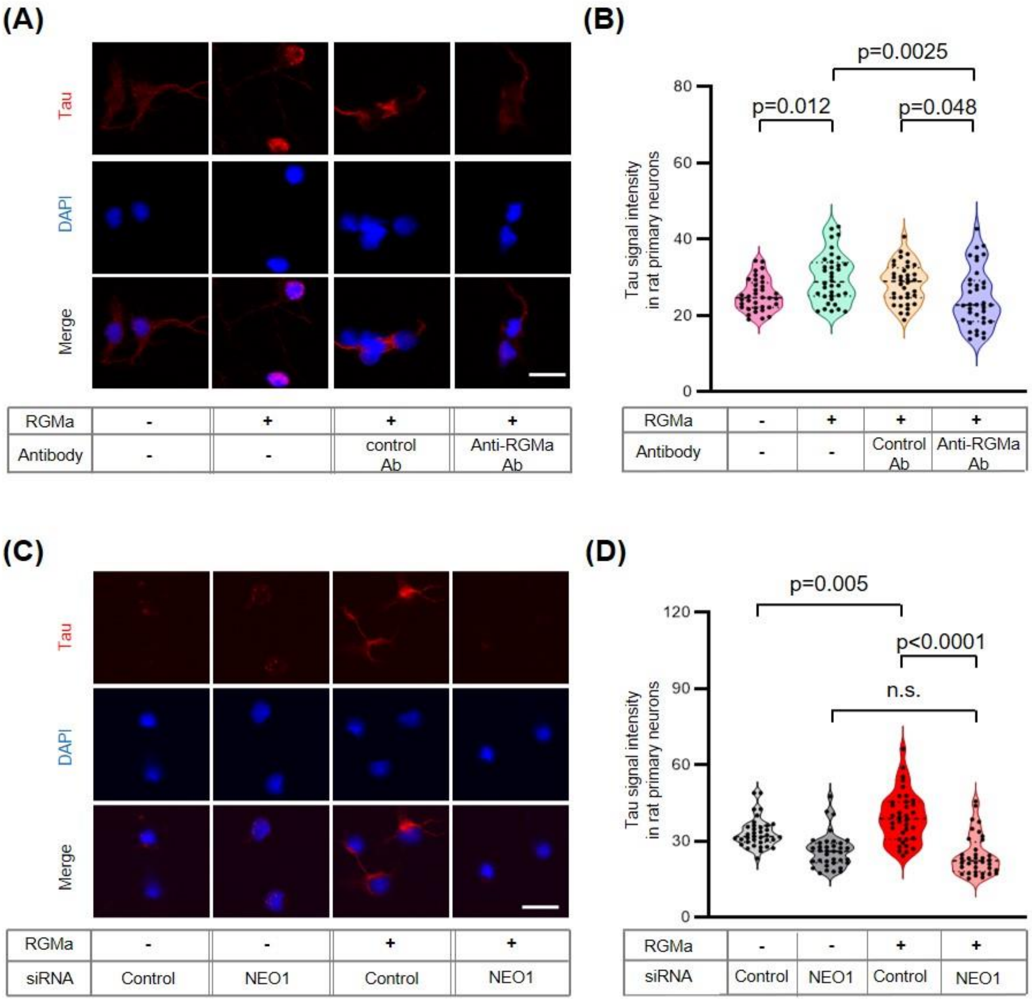

Fig. S6. Anti-RGMa antibody or gene silencing of NEO1 suppressed the entry of rTau protein into rat primary cortical neurons in vitro.

(A) Recombinant tau proteins were administered to rat primary neurons treated with or without RGMa and incubated with anti-RGMa or control Abs. Recombinant tau proteins were visualized using an anti-tau antibody conjugated with Alexa Fluor^{TM}594. Representative images are shown above. **(B)** The mean fluorescence signal intensity of the rTau protein in Figure S6A is quantified. **(C)** Rat primary neurons were treated with or without RGMa after NEO1 silencing. Recombinant tau was administered and subsequently visualized using an anti-tau antibody via immunofluorescence. Representative images are shown above. **(D)** The mean fluorescence signal intensities in Figure S6C are quantified. Each filled dot represents the mean fluorescence intensity of a single cell. Under all the conditions, 36 cells in 2 individual wells were selected. Scale bar, 20 µm. Error bars indicate the mean ± SEM.

Ab, antibody; RGMa, repulsive guidance molecule A; NEO1, Neogenin 1; siRNA, small interfering RNA; rSOD1, recombinant superoxide dismutase 1; rTau; recombinant tau; WT, wild-type; SEM, standard error of the mean.

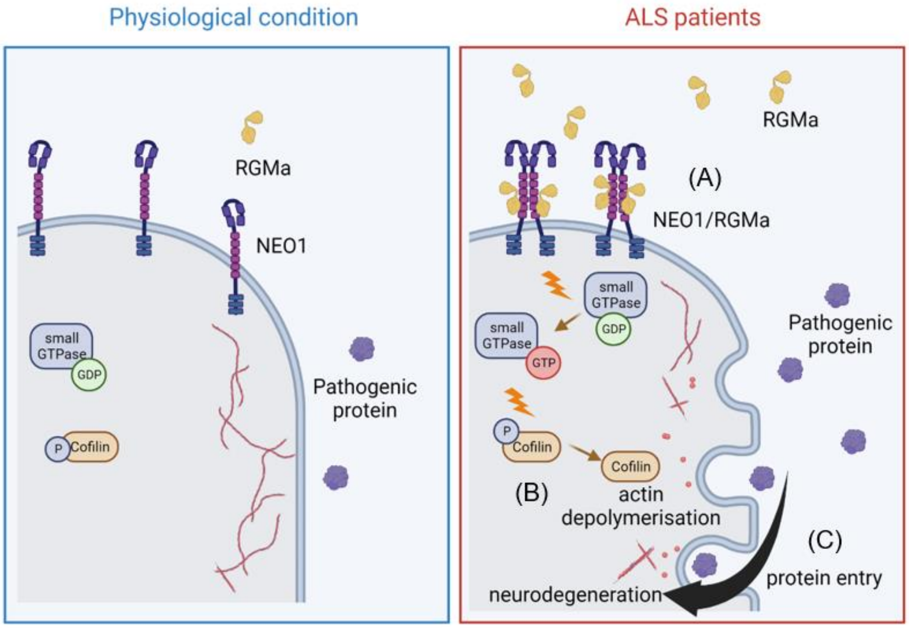

Fig. S7. The RGMa/NEO1 axis plays a pivotal role in promoting pathogenic proteins uptake via the collapse of the actin barrier.

(A) The RGMa ectodomain shed from the cell surface binds to pre-clustered NEO1, leading to stabilization and dimerization of the NEO1 ectodomain, and subsequently activates downstream signaling, such as the small GTPase cascade. (B) The small GTPase cascade dephosphorylates cofilin and enhances actin depolymerization. (C) Actin depolymerization induces the collapse of the actin barrier and promotes the entry of extracellular pathogenic proteins, such as mutant SOD1, leading to an exacerbated neuronal degeneration.

RGMa, repulsive guidance molecule A; NEO1, neogenin1; GTP, guanosine triphosphate; ALS, amyotrophic lateral sclerosis; SOD1, superoxide dismutase.

Table S1. Clinical characteristics of participants

The levels of RGMa in the CSF of ALS, NDs, and ONDs were shown below. And number, sex and age of patients were also indicated.

			(mean ± STD)	
	Number of patients	Male/Female	Age	RGMa (ng/mL)
ALS	30	19/11	63.4±12.6	9.43±2.96
NDs	24	14/10	62.7±15.9	6.79±2.80
ONDs	40	19/21	63.1±18.2	5.67±2.75

RGMa, repulsive guidance molecule A; STD, standard deviation; ALS, amyotrophic lateral sclerosis; NDs, non-neurodegenerative disorders; ONDs, other neurodegenerative disorders; CSF, cerebrospinal fluid

Table S2. Demographics of ALS patients in whom CSF RGMa concentrations were measured

Demographic characteristics of ALS patients were shown below.

	Patients with ALS (n=30)		
	Definite (n=6)	Probable (n=17)	Possible (n=7)
Age (years)	68 ± 13.3	57.8 ± 10.8	73 ± 7.43
Male/Female	5/1	7/10	7/0
ALSFRS-R	39.8 ± 2.11	42.6 ± 2.81	43.1 ± 2.1
ΔFS	0.668 ± 0.31	0.314 ± 0.18	0.479 ± 0.25
%VC	86.9 ± 16.4	95.2 ± 16.9	82.2 ± 17.3
$PaCO_2$ (mmHg)	38.7 ± 3.92	42.6 ± 4.21	40.1 ± 2.26
RGMa (ng/ml)	8.09 ± 2.3	10.0 ± 3.02	9.16 ± 2.89

Plus-minus values are means ± STD. CSF, cerebrospinal fluid; RGMa, repulsive guidance molecule A; ALS, amyotrophic lateral sclerosis; ALSFRS–R, ALS Functional Rating Score Revised; ΔFS, (48 – ALSFRS-R Score at Time of Diagnosis)/Duration From Onset to Diagnosis; VC, vital capacity; $PaCO_2$, partial pressure of arterial CO_2

Table S3. Age at death, sex, postmortem interval, and pathological diagnosis of postmortem cases

Demographic characteristics of the postmortem cases were shown below.

Case No.	Age at death (y)/Sex	Postmortem Interval (hrs)	Pathological Diagnosis
Controls			
1	68/M	7	Multiple cerebral infarction
2	69/M	23	Pontine stroke
3	74/M	4	Intracranial hemorrhage
ALS			
1	77/F	40	ALS
2	63/M	2	ALS
3	77/M	9	ALS

ALS, amyotrophic lateral sclerosis; NEO1, neogenin1; y, years; hrs, hours; M, male; F, female
